# Safety and efficacy of anti-hyperglycemic agents in patients with type 2 diabetes mellitus (T2DM): Protocol for an overview of systematic reviews based on network meta-analysis

**DOI:** 10.1371/journal.pone.0282143

**Published:** 2023-03-03

**Authors:** Zhengping Chang, Jianguo Xu, Yu Qin, Qingyong Zheng, Liang Zhao, Yunfang Wang, Yan Zhang

**Affiliations:** 1 Department of General Medicine, Pingliang Rehabilitation Center Hospital, Pingliang, China; 2 Evidence-Based Medicine Center, School of Basic Medical Sciences, Lanzhou University, Lanzhou, China; 3 Evidence-Based Nursing Center, School of Nursing, Lanzhou University, Lanzhou, China; 4 Department of Endocrinology, Gansu Provincial Hospital, Lanzhou, China; 5 Department of Spinal Cord Injury Rehabilitation, Gansu Province Hospital Rehabilitation Center, Lanzhou, China; Dilla University, ETHIOPIA

## Abstract

Type 2 diabetes mellitus (T2DM) has caused a huge clinical and economic burden worldwide. The management strategy of T2DM has been mentioned in many guidelines. However, controversy still exists in the recommendation of anti-hyperglycemic agents. To this end, this protocol has been written according to the Preferred Reporting Items for Systematic Reviews and Meta-Analyses Protocols (PRISMA-P). We will make an overview of systematic reviews based-on network meta-analysis firstly that report on safety and efficacy of different category of anti-hyperglycemic agents for T2DM patients. We will identify network meta-analysis by applying a robust and standardized search strategy within Embase, PubMed, Web of Science, and Cochrane Database of Systematic Reviews. Hemoglobin A1c (HbA1c) and fasting plasma glucose (FPG) will be defined as the primary outcomes. We will assess the methodological quality of included reviews by applying the A MeaSurement Tool to Assess Systematic Reviews (AMSTAR-2) tool, and quality of evidence for all outcomes will be judged by using the Grading of Recommendations Assessment, Development and Evaluation (GRADE). This will provide an accessible narrative synthesis to clinicians, patients, policy makers, and developers of clinical guidelines based on published high-quality network meta-analysis. We will submit our results for peer-review publication and presentation at domestic and international conferences. We will also disseminate our results through established clinical networks and consumer networks, using pamphlet where appropriate. Ethics approval is not required for this overview as we will analysis published network meta-analysis only.

**Trial registration number:** INPLASY202070118.

## 1. Introduction

Diabetes is a chronic and progressive disease featured by the deterioration in blood glucose control over time. It has caused significant clinical and economic burdens worldwide [1]. Twenty years ago, an estimated 151 million adults worldwide had diabetes. A decade ago, in 2010, the incidence of diabetes increased by 88% to 285 million, and now this data is 463 million [2,3]. If adequate action is not taken to address the pandemic, The International Diabetes Federation (IDF) estimates that there will be 578 million adults with diabetes by 2030, and 700 million by 2045 [3]. In 2019, the worldwide total diabetes-related healthcare expenditure for adults aged 20–79 are estimated to be USD 760 billion, of which the majority (68.7%) are those aged 50–79 years. It is estimated that by 2030, related healthcare expenditure will grow to USD 825 billion [3,4].

Type 2 diabetes mellitus (T2DM) is the most common type of diabetes, accounting for approximately 90% of diabetes worldwide. Globally, the prevalence of T2DM is estimated at 9% in the adult population, and it is rising across all regions [5,6]. This rise is driven by an aging population, economic development, and growing urbanization. The foundation of T2DM management is to maintain a healthy lifestyle and an appropriate body weight [7]. If trying to change lifestyle is not enough to control blood glucose levels, current guidelines [8] recommend metformin as the first-line agent for the treatment of T2DM with insufficient diet and exercise. When metformin monotherapy cannot be tolerated or contraindicated, or the efficacy is insufficient to control hemoglobin A1c (HbA1c) to achieve the desired target, the second anti-hyperglycemic agent is recommended as an alternative or additional therapy [9,10]. When oral medications are unable to control hyperglycemia to recommended levels, insulin therapies may be necessary [11]. However, the recommendations of different guidelines [8–10,12,13] regarding the selection of second-line agents are also controversial. Risk stratification management and therapeutic regimens optimization of T2DM patients need high-quality evidence to support it.

In 2011, the *Ann Intern Med* published the first network meta-analysis [14] on the comparative effectiveness of glucose-lowering drugs for T2DM. In the following ten years, many similar network meta-analysis appeared in this domain, comparing the safety and efficacy of different combination, dosage, course of treatment, and frequency of medication on T2DM between different anti-hyperglycemic drugs [15–18]. This overview will formally assess the quality of methodology and evidence of existing network meta-analysis in this domain.

Specifically, the objectives we aim to access include: 1) make a narrative synthesis of the evidence to get an insight for different agent therapy strategy for T2DM, and 2) make an evidence map based on Grading of Recommendations Assessment, Development and Evaluation (GRADE) to guide the use of anti-hyperglycemic agents. To this end, the proposed overview of systematic reviews will answer the following questions: 1) What is the methodological quality of published network analysis in this field? 2) What are the efficacy and safety of various anti-hyperglycemic agents verified by systematic review based on network meta-analysis and which agents are suitable for patients with different risk stratification? 3) Based on GRADE hierarchy of evidence, what are the strength of evidence and recommendation level of the various agents included in the network meta-analysis?

## 2. Materials and methods

### 2.1. Protocol and registration

In accordance with the guidelines, the protocol for this overview of systematic reviews was reported with the Preferred Reporting Items for Systematic Review and Meta-Analysis Protocols (PRISMA-P) 2015 checklist [19,20]. It was registered with the International Platform of Registered Systematic Review and Meta-analysis Protocols (INPLASY) [21] on 27 July 2020 and was last updated on 5 August 2022 (registration number INPLASY202070118). Available in full on the inplasy.com (https://doi.org/10.37766/inplasy2020.7.0118).

### 2.2. Eligibility criteria

Studies will be selected based on the criteria listed below to identify multiple systematic reviews on related research questions in the same topic area [22]. If the research protocol in PICOS is revised, the date of each amendment about eligibility criteria will be accompanied by an explanation of the changes and reasons.

#### 2.2.1. Study designs

We will include peer-reviewed and published network meta-analysis of anti-hyperglycemic agents for T2DM which provide meta-estimates for outcomes. Both direct comparison and indirect comparison of network meta-analysis will be included. The network meta-analysis that is ongoing or published in the form of conference abstracts will not be included.

#### 2.2.2. Participants

We will limit our overview of systematic reviews to studies of adults with T2DM, regardless of gender, race, or the presence of insulin resistance. The pregnant women with gestational diabetes will be excluded.

#### 2.2.3. Interventions/Comparators

Comparisons among the following interventions were included: insulin, metformin, sulfonylureas, thiazolidinediones (TZDs), active comparator drugs (ACDs), dipeptidyl peptidase-4 (DPP-4) inhibitors, Glucagon-like peptide-1 (GLP-1) analogues or agonists, sodium/glucose cotransporter 2 (SGLT-2) inhibitors, α-glucosidase inhibitors, meglitinides, or placebo. There are no restrictions on the combination formula such as whether to plus other agents to metformin or sulfonylureas. There are also no restrictions on different doses or frequency of the same agent. We classify all eligible drugs according to the above drug categories and because different drugs in the same category may have a variable effect, we include studies that compare drugs in a same category either. If a network meta-analysis included drugs of interest but also included drugs that were not of interest, or if multiple interventions included glycemic control by non-pharmacological methods, such studies would also be included. Interventions includes some drugs but not any drugs of interest within the list except for comparator drugs will not be included.

#### 2.2.4. Outcomes

The primary outcomes are HbA1c and fasting plasma glucose (FPG). The second outcomes are body mass index (BMI), 2 h postprandial blood glucose (2HPPG), body weight and adverse events, including hypoglycemia, diarrhea, upper respiratory tract infection (URTI), hypersensitivity reaction (HR), cardiovascular outcomes, renal and hepatic toxicity [23,24]. The second outcomes will be adjusted according to the final inclusion of the literature.

#### 2.2.5. Language

No restrictions will be placed on the original languages to which the literature will be included. Languages other than English and Chinese will be processed with the help of translation software tools or by seeking native speakers.

### 2.3. Information sources and search strategy

Our overview will search for systematic reviews including network meta-analysis from the following databases: PubMed, Embase, Web of Science, and Cochrane Database of Systematic Reviews. Search terms include *diabetes* and *network meta-analysis*. A researcher from evidence-based medicine center will create and run a search string to identify relevant articles. Take electronic databases PubMed which planned to be searched as an example, the pre-search strategy is presented in **Table 1**. The search strategy will undergo internal peer review. The research is expected to officially start in December 2022.

**Table 1:** PubMed search strategy.

PubMed	PubMed Query
**#1**	(("Diabetes Mellitus"[Mesh]) OR (diabetes))
**#2**	("Network Meta-Analysis"[Mesh])
**#3**	("network meta analysis" OR "network meta analyses")
**#4**	("mixed treatment comparison meta analysis" OR "mixed treatment comparisons meta analyses")
**#5**	("mixed treatment meta analysis" OR "mixed treatment meta analyses")
**#6**	("mixed treatment comparisons" OR "mixed treatment comparison")
**#7**	("multiple treatment comparison meta analysis" OR "multiple treatment comparisons meta analyses")
**#8**	("multiple treatments meta analysis" OR "multiple treatments meta analyses")
**#9**	("multiple treatment meta analysis" OR "multiple treatment meta analyses")
**#10**	("multiple treatment comparison" OR "multiple treatment comparisons")
**#11**	#2 OR #3 OR #4 OR #5 OR #6 OR #7 OR #8 OR #9 OR #10
**#12**	#1 AND #11

### 2.4. Study records

#### 2.4.1. Data management

The retrieved articles from the databases were exported to EndNote X9 for duplicate removal and further categorization. The full text of eligible reviews will also be attached to EndNote X9.

#### 2.4.2. Network meta-analysis selection and data collection

We will follow the recommendations in the Cochrane handbook for quality control and transparency of independent screening [25]. Two authors (one is a physician in the Department of Endocrinology, and the other is a researcher from the Evidence-Based Medicine Center) will independently screen the titles and abstracts, than to determine the preliminary inclusion of systematic reviews according to the eligibility criteria. While insufficient data are available or where there is any uncertainty in the abstract, the full-text will be retrieved. Any differences in selection will be resolved through discussion to reach a consensus or by adjudicating with a third author. We will record the excluded articles and the reasons for their exclusion. If necessary, we will get additional information for unclear or doubtful data from the corresponding authors by email. We shall use Microsoft Excel to perform pre-development spreadsheets to extract data of each review. The third author will check the data extracted by the two reviewers, and finally reach a consensus on the inconsistent data through discussion.

### 2.5. Data items

The reviewers will extract the following data items from each included systematic review: 1) Bibliographic details (author, institution, publication year, journal, country, funding). 2) Methodological characteristics (search end date, study design of primary research, agent and dose, length of duration of treatment, funding). 3) Method of pooling and bias assessment (homogeneity assumptions, similarity assumptions, and consistency assumptions in network meta-analysis, the choice of frequency and Bayesian methods in statistics, and the choice of statistical software for analysis to refine our study, risk of bias assessment tool). 4) Patient characteristics (age, gender, race, the therapeutic effect of first-line drugs). 5) Results (number of studies included in meta-estimate, event rate in different arms or patient populations, meta-estimate, risk of bias within included studies, risk of bias in meta-estimate).

### 2.6. Risk of bias individual studies

#### 2.6.1. Assessment of methodological quality of included reviews

Two reviewers will independently assess the methodological quality of included network meta-analysis using A MeaSurement Tool to Assess Systematic Reviews (AMSTAR-2) tool [26]. The AMSTAR-2 has 16-domains covering topics including review registration, comprehensiveness of the literature search, inclusion/exclusion strategy, critical appraisal/results synthesis, and risk of bias (e.g., assessment and publication bias). Each domain is rated ‘yes’, ‘partial yes’, or ‘no’, and the overall quality of the study will be rated as ‘high’, ‘moderate’, ‘low’, or ‘critically low’ [27]. Any differences between author assessments will be resolved by discussion or adjudication by a third author. We will not exclude any reviews from the overview based on the results of this assessment.

#### 2.6.2. Assessment of quality of evidence

The quality of evidence for all outcomes will be judged using GRADE systems [28,29]. This tool has been previously selected and applied to improve the transparency and consistency in quality assessments of overview of systematic reviews [30,31]. The quality of evidence will be assessed across the domains of risk of bias, consistency, directness, precision, and publication bias. Quality will be adjudicated as high, moderate, low, or very low. Two principles of the original GRADE NMA guidelines are that we need to rate the certainty of each pairwise comparison of evidence within the network individually, and that in doing so we need to consider both direct and indirect evidence [32]. We follow the GRADE group’s recommendations for assessing the certainty of evidence: (1) it is not necessary to consider imprecision when rating direct and indirect estimates in order to inform the rating of NMA estimates; (2) it is not necessary to rate indirect evidence when the certainty of the direct evidence is high and the contribution of the direct evidence to the network estimates is at least as large as the contribution of the indirect evidence. (3) we should not trust statistical tests of the overall inconsistency of the network to assess inconsistency at the pairwise comparison level, and (4) where there is inconsistency between direct and indirect evidence, the certainty of the evidence for each estimate can help in deciding which estimate to trust.

The strict criteria on which we will base our synthesis will ensure that only those systematic reviews based on network meta-analysis with a high quality contribute to the evidence [33]. Disagreements over the assessment of the quality of evidence will be resolved by discussion with a third author.

### 2.7. Data synthesis

We will consider the issue of overlapping primary studies prior to preparing our evidence synthesis. If there are multiple network meta-analysis that include the same agents in the same patient, and measure the same outcome, we will deal with overlapping primary documents by the following methods: 1) If the primary research completely overlaps (repetition rate ≥ 80% of any one of the article), then we will choose the highest quality review.2) If the primary studies partially overlap, and the repetition rate is between 50% and 80%, then we will retain both reviews if the lower-quality review consists of more than 30% new studies. 3) If the primary studies do not overlap (repetition rate ≤ 50%), then we will retain both reviews [34].

We will present the findings as a narrative synthesis, and will take tabulated summaries and visualized evidenced map to display the data [35]. The presentation of results will follow a simple visual ’traffic light’ indicator, where green indicates that the intervention is beneficial, orange indicates that there is no difference in the comparison of the surveys, and red indicates that the results indicate that the intervention is harmful or less effective than the controls [36]. When results are not reported in the network meta-analysis, the indicator box is left blank. We will give recommendations for clinical use of agents based on evidence-based medical evidence. We will consider evidence to be sufficient if a systematic review is of high quality. On the contrary, we will not compute an overview meta-estimate due to the likelihood of considerable heterogeneity in study populations and outcomes between studies, the absence of essential meta-data and the lack of well-established quantification methods. Results of this overview will be reported according to the Preferred Reporting Items for Systematic Reviews and Meta-Analyses (PRISMA) guidelines [37]. The reasons for any amendments of protocol will be documented in the full review.

## 3. Discussion

This study will be the first overview of systematic reviews based on network meta-analysis. We will use rigorous methodology to seriously and systematically appraise and synthesis published systematic reviews with network meta-analysis evidence. The results of the many network meta-analysis overwhelm readers, and we hope to be able to summarise and sort them out further for users of the evidence. It is a great ambition to perform a secondary combined analysis of the data from the numerous network meta-analyses, and our team did not consider a combined analysis of the data in this work. Based on the published network meta-analysis, the efficacy and safety of various anti-hyperglycemic agents will be systematically reviewed and validated, and used GRADE to validate the quality of evidence from different studies to identify the most suitable treatment options for T2DM patients in different risk strata. We will provide new commentary and higher levels of evidence for the harm and benefits associated with diabetes medications [38]. The overview of reviews will provide a more comprehensive and integrated evidence-based opinion for guideline development, and make the rational use of drugs for T2DM patients more transparent and reliable. We expect the results of this comprehensive synthesis overview base-on network meta-analysis will benefit physicians, policy makers and developers of clinical guidelines for the management of T2DM patients with different risk stratifications. Furthermore, we also expect this overview of systematic review will improve the quality of secondary research in this field in the future and encourage more original research for those agents for which evidence is controversial or lacking in the current systematic reviews.

## Supporting information

S1 Checklist PRISMA-P (Preferred Reporting Items for Systematic review and Meta-Analysis Protocols) 2015 checklist: Recommended items to address in a systematic review protocol*. (DOC)

## Abbreviations

T2DM: Type 2 diabetes mellitus
IDF: International Diabetes Federation
INPLASY: International Platform of Registered Systematic Review and Meta-analysis Protocols
TZDs: thiazolidinediones
ACDs: active comparator drugs
DPP-4 inhibitors: dipeptidyl peptidase-4 inhibitors
GLP-1: Glucagon-like peptide-1
SGLT-2 inhibitors: sodium/glucose cotransporter 2 inhibitors
HbA1c: hemoglobin A1c
FPG: fasting plasma glucose
2HPPG: 2 h postprandial blood glucose
BMI: body mass index
URTI: upper respiratory tract infection
HR: hypersensitivity reaction
PICOS: Participants, Intervention, Control, Outcome, Study design
GRADE: Grading of Recommendations Assessment, Development and Evaluation
AMSTAR: A MeaSurement Tool to Assess Systematic Reviews
PRISMA: Preferred Reporting Items for Systematic Reviews and Meta-Analyses
